# Review of Cementitious Composites Containing Polyethylene Fibers as Repairing Materials

**DOI:** 10.3390/polym12112624

**Published:** 2020-11-07

**Authors:** Shuai Zhou, Lina Xie, Yue Jia, Chong Wang

**Affiliations:** College of Materials Science and Engineering, Chongqing University, Chongqing 400045, China; 201909021017@cqu.edu.cn (L.X.); 20162802@cqu.edu.cn (Y.J.)

**Keywords:** polyethylene, engineered cementitious composites, repair

## Abstract

Polyethylene (PE) is an important polymeric material which is widely used in civil engineering. Recently, engineered cementitious composites (ECCs) have adopted PE fibers in structural repairing. ECC with polyethylene fibers (PE-ECC) has excellent tensile properties, ductility, strain-hardening behavior, thermal performance and durability. In this paper, a systematic review of the cementitious composites with PE fibers is summarized to facilitate the application of PE-ECC. The influence of PE fibers on the properties of ECC, such as compressive strength, flexural behavior, shear properties, impact resistance and tensile properties, is presented. Meanwhile, the properties of PE-ECC repaired structures, such as beams, walls and columns, are described. Further, the self-repairing properties of PE-ECC are presented. Finally, some suggestions for future research are provided in order to apply PE-ECC to practical repairing cases. The review exhibits that PE-ECC is of notable significance to the repairing of structures and clarifies its application scope.

## 1. Introduction

**Figure 1:**
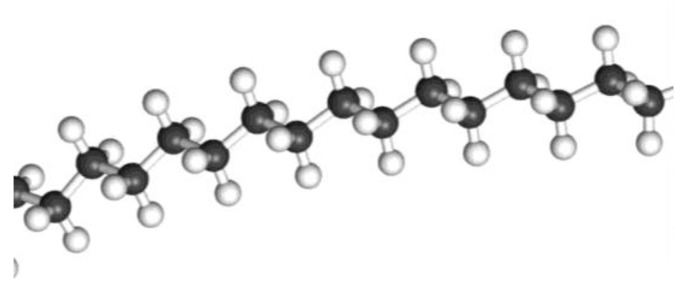
The molecular structure of polyethylene (PE).

Polyethylene (PE) is a significant material and has been applied in composites since it was developed [1,2]. It is made from the polymerization of ethylene and is a member of the important family of polyolefin resins. As a thermoplastic polymer, PE has outstanding toughness, abrasion resistance, impact resistance and low water absorption with low costs [3]. Compared with other commercial polymeric materials, PE is the second most heavily used polymer [4]. PE has a simple molecular structure, as is shown in Figure 1.

PE has been widely used in civil engineering for a long time [5]. Raupach and Morales Cruz [6] developed the two-layer structure of PE and textile-reinforced concrete in pressure pipes, pressure-free pipes, tunneling pipes or the corrosion protection of steel pipes. PE fibers were applied in 3D printable cementitious composites [7,8,9,10]. PE can be used as the coating material to produce PE coated bars in concrete [11] and composite rods as steel bars [12]. PE fibers were adopted in the geopolymer composites as the reinforced material [13]. It can act as the matrix to repair structures together with basalt fibers, which have a high paste strength [14]. Zhuang and Zhou [15] considered the usage of PE in concrete and studied the interfacial properties between the cementitious matrix and PE molecules.

PE fibers have good chemical resistance, tensile strength, easy production process and many other excellent properties. The molecular weight and degree of branching influence the physical properties greatly. PE fibers contain two types: low-density polyethylene fibers and high-density polyethylene fibers. Li and his coworkers firstly added PE fibers into cementitious materials and developed Engineered Cementitious Composites (ECC) [16]. Concrete is a kind of brittle material. By adding PE fibers in it, the cementitious composites show the ductility. ECC is a kind of fiber-reinforced cementitious material with ductility and strain-hardening performance [17]. It is effective in civil engineering, like bridges [18,19], road pavements [20,21,22,23,24,25,26], nuclear power plants [27], masonry structures [28] and concrete buildings [29,30,31,32,33,34].

Concrete structures are exposed to earthquakes, extreme weather or erosive conditions. It is inevitable for them to experience damage and fracture. Sulfate attack, chloride ingress and mechanical damage are severe threats for cementitious materials [35]. They may result in severe degradation and disasters for civil engineering structures, causing possible serious casualties and property losses. Hence, their repair is vital. The repair of aged civil structures attracts more and more attention recently. In order to handle the problem, new technology and materials are wanted. Many researchers have investigated the behavior of repaired structures by PE-ECC since its invention [36]. 

The feasibility, cost and application simplicity of the method should be considered in the selection of appropriate retrofitting methods. Concrete jacketing is a mature and convenient solution for repairing, retrofitting and strengthening existing structures. It can improve flexural/shear bearing capacity, deformation capability and stiffness of concrete members. It provides a protective layer against corrosion of the internal reinforcement. Hence, it is a good retrofitting method for structures in marine and coastal environments compared with fiber reinforced polymer (FRP) wrapping and steel jacketing. However, the traditional concrete jacketing method has some disadvantages, such as the increased dead load and the reduced available space of structures. In addition, the durability problems associated with concrete shrinkage exist in the structures retrofitted by the traditional concrete jacketing method, since additional stresses will be generated in the retrofitted concrete which lead to cracking and peeling of the jacket. Therefore, in order to effectively reduce the crack width and improve the durability of core concrete, it is necessary to find an alternative material as the jacketing layer. With the development of concrete jacketing technology, a jacket made by ECC is considered to be a promising alternative to the traditional concrete jacket in concrete structure retrofitting. The ECC jacket has greater strain capacity than the ordinary concrete jacket. Meanwhile, since the porosity and the permeability of ECC are lower than those of ordinary concrete, it can effectively prevent the corrosion of internal reinforcements and the carbonization of core concrete. This characteristic makes it an ideal choice for retrofitting concrete members subjected to large inelastic deformation and harsh environments. At the same time, because FRPs cannot effectively strengthen beam–column joints, the jacket of undamaged or cracked reinforced concrete members has advantages. Compared with the initial beam, the shear strength and deformation capacity of the ECC jacketed beam are improved. The strengthened beams have lower brittleness and higher deflection. The failure mode of retrofitted beams can be changed from brittle failure to ductile failure [37]. Compared with the traditional concrete jacket, the ECC jacket has superior mechanical properties. Compared with FRP wrap and steel jacket, ECC has higher durability advantages. Therefore, ECC has great potential as a high-performance material in structural retrofitting [38].

With the development of concrete jacketing techniques, thin fiber-reinforced concrete jackets made with ECC have been considered a promising alternative to the conventional concrete jacket in the retrofit of concrete structures. The repairing techniques were conveniently implemented. Firstly, the surfaces of the specimen around the damaged area were ground using an electric grinder to ensure smooth contact surfaces. After that, they were cleaned by using air blasting before bonding and wrapping with ECC. It can be in the form of shotcrete or cast in situ.

PE-ECC can reduce brittle properties and improve tensile performance. The tensile strain capacity is about 3–7%, while plain concrete breaks at 0.01%. The tensile strength, shear strength, flexural strength, deflection capacity, ductility, toughness and energy dissipation ability of ECC or ECC structures can be enhanced greatly. Since it can saturate small voids of existing concrete, it is wildly used to repair the concrete structures. Further, it can dissipate the energy by generating multiple cracks, which stops the structure from collapsing suddenly under earthquakes. However, ECC is more expensive than plain concrete due to the usage of PE fibers. Hence, it should be designed properly before using it.

The properties of cementitious materials with PE fibers are summarized in this research to apply these characteristics to civil engineering structures. Section 2 reported the related properties of PE-ECC. The performance of repaired structures by PE-ECC was analyzed in Section 3. In Section 4, the self-repairing capacity of PE-ECC was concluded. Finally, the conclusions of the present study were summarized in Section 5. 

## 2. The Properties of Cementitious Materials with PE Fibers

### 2.1. Physical Properties

There are many literatures about the physical properties of PE fibers, which were listed in Table 1 [39,40,41].

The production process of PE-ECC is now highly mechanized [39,40,41]. All mixtures (e.g., sand, cement, fly ash, ground granulated blast furnace slag, silica fume, water reducer, fibers) were prepared in a mixer. The solid dry raw materials were mixed all together for 2 min. Subsequently, water and water reducer were added and mixed to reach a proper fluidity of the matrix. Finally, fibers were added slowly into the mortar by hands and mixed for 3 min to ensure good dispersion. The fresh mixture was cast into steel molds covered with plastic sheets and demolded after one day curing. All the specimens were cured in air for a period of time before testing.

Previous research revealed that PE fibers decreased the slump of cementitious materials. PE fibers reduced the slump, which led to uneven fiber distribution. The cementitious composites had trouble in homogeneity and workability [30]. PE fibers increased the volume fraction of air voids. The air void increased from 0.4% to 1.2% when the PE fibers were incorporated. Our research group is developing an innovative vibration-aided method to solve the problem. The results show that the new mixing method is conducive to the reduction of air void and the dispersion of PE fibers [42]. The fibers under vibration can be distributed more uniformly.

The addition of PE fibers can reduce the drying shrinkage of cementitious materials. More than 10% reduction of the drying shrinkage of cementitious materials was witnessed [30]. The crack width and crack area decreased with the content of PE fibers [43].

### 2.2. Static Mechanical Properties

#### 2.2.1. Tensile Performance

It is known that plain concrete exhibits brittle collapse when the developed tensile stress exceeds the limited strength of concrete under tension. The use of non-conventional mass reinforcement such as discontinuous fibers has been proven to be a promising alternative. 

Maalej et al. [44] investigated the influence of the volume fraction of PE fibers on the tensile properties of cementitious composites. The results proved that with the increase of volume fraction of PE fibers, the off-crack-plane fracture energy increased. When the volume fraction of PE fibers was 4%, the fracture energy was up to 34 kJ/m^2^. The volume fraction of PE fibers should be above 0.4% to achieve damage-tolerant behavior.

Hybrid fibers (steel and PE fibers) were added into cementitious materials and investigated by Ahmed and Maalej [45]. PE fibers increased the tensile strain capability, while steel fibers improved the tensile strength of cementitious composites. Longer PE fibers could raise the tensile strain capacity and strain-hardening behavior of cementitious materials. Multiple cracks were observed in the research. Sand in ECC had a negative influence on the tensile strain ability.

The tensile tests of the cementitious composites with PE fibers were conducted. Good stain hardening behavior was reported [46]. Meanwhile, multiple fine cracks were produced, which was significantly different from normal concrete.

Hybrid fibers were considered in previous research. When PE fibers and steel fibers were adopted together in cementitious materials, the material showed higher first crack initiation stress, ultimate tensile strength and tensile strain capacity, which can be used to improve the cementitious composite [47].

PE fibers were added into concrete, which can be used to repair the dam hydraulic surfaces. PE-contained concrete had the greatest tensile strength compared with fiber-free concrete when the content was 2.5%. Further, it showed better abrasion resistance [48].

Tensile tests of PE-ECC showed the ductile behavior and the strain hardening performance of PE-ECC. The shape of PE fibers influenced the tensile properties of PE-ECC. When D = 10 and aspect ratio = 316, ECC had the maximal tensile strength with 1.5% as the PE fiber content. When D = 4 and aspect ratio = 500, 1% is the optimal dosage [49].

The tensile performance of ECC was examined when PE and steel fibers were mixed in the cementitious materials. Energy dissipation and deformation behavior rose when steel fibers were replaced by PE fibers. The best usage volume was 2% fiber volume fraction. The energy absorption capacity was much greater than the specimens with only steel fibers [50].

The average tensile strength increased to 14.5 MPa and the average tensile strain went up to 3.4% when PE fibers were incorporated in ECC in previous research [51]. The average tensile elastic modulus of ECC was 48.4 GPa. The tensile results showed great ductility. 

PE-ECC had a large tensile strength of 17.42 MPa with a coefficient of variation of 6%. Meanwhile, the tensile strain reached 8.17% with a coefficient of variation (COV) of 5% with the volume fraction 2% [52]. Multiple cracks appeared as the loading increased. Localized cracking did not occur. The crack width was less than 100 μm, which illustrated the low permeability.

PE fibers were utilized in PE-ECC together with steel fibers. The hybrid ECC had a good tensile property, ductility and fracture performance compared with plain concrete. The fiber orientation of steel fibers was influenced by PE fibers, which influenced the tensile properties [53]. 

Zhou et al. [54] applied PE fibers and fly ash to manufacture lightweight ECC. It had the advantages of ECC, like the strain hardening effect and good tensile properties. Further, the density was lower than the normal ECC. The normal ECC reached 2250 kg/m^3^, while the value was just 1380 kg/m^3^ in the lightweight ECC. The ductility of PE-ECC did not decrease even the flexural strength reduced due to the fly ash.

He et al. [55] developed carbon nanofibers-coated PE fibers and used it in cementitious materials. The interface transition zone (ITZ) between the matrix and PE fibers was enhanced. ECC with carbon nanofibers-coated PE fibers was developed. Tensile strength increased by 10%. Tensile strain capacity rose by 20%. Meanwhile, the interfacial bond strength of the cementitious matrix and CNF-coated PE fibers increased by 22%. The carbon nanotube can strengthen the ITZ by filling nano-pores and bridging cracks. Stronger ITZ meant greater tensile strength and strain ability.

The tensile strength and tensile strain of PE-ECC with 1.5% PE fibers after 100 and 200 cycles of freezing and thawing reduced compared with the reference sample. The deformation capacity of PE-ECC dropped greatly after 200 cycles, while it was still greater than 0.5% [56]. 

ECC with PE and steel fibers was tested under tensile loading [57]. The PVA-ECC specimens were tested at the same time. The results showed that multiple microcracks were generated all over the specimen in both cases. PE and steel fibers were not broken. The bridging effect still worked between the crack surface. However, the PVA fibers were ruptured due to the low strength compared with PE fibers. It was concluded that PE fiber composites showed better performance than PVA fiber composites.

Deng [58] developed high-early-strength ECC using PE fibers. Sulfoaluminate was used instead of Portland cement as the binding material. Within 6 h, the high-early-strength ECC had a tensile strength of more than 2.5 MPa and a tensile strain capacity of 3%. At 60 days, tensile strength of 5 MPa and ultimate tensile strain of more than 3.5% were obtained in the research.

Yu et al. [59] adopted the high-molecular and high-strength PE fibers in the cementitious materials to develop the ultra-high ductile cementitious composite. The crack spacing of PE-ECC reached 2 mm. Meanwhile, the crack width achieved 100 micrometers. The tensile strain was up to 8–12%. The ultra-high ductility came from the crack bridging ability. The research concluded that if fibers were strong enough, ECC can achieve super ductility.

Hybrid fibers (steel and PE fibers) were added into cementitious materials to produce ECC. The uniaxial tension test was carried out. PE fibers proved to be effective in enhancing the strain-hardening effect and strain ability. Multiple cracks were observed in the experiments. Steel fibers can increase the strength. Hence, the hybrid fiber system had great potential in cementitious materials [60]. A similar combination was considered by Choi et al. Improved ductility was witnessed. Different diameters influenced the bond strength and energy dissipation [61]. 

Yun et al. [62] adopted PE, PVA and steel fibers in cementitious composites. The combination of fibers increased the tensile strength and crack initiation stress. Ductility and energy-absorbing ability rose.

Plasma treatment of PE fibers was investigated by Li et al. [63]. Fiber reinforcements were influenced greatly by the surface condition of PE fibers. Meanwhile, the load transfer from fibers to the cementitious matrix was associated with the ITZ, which determined the properties of ECC. The plasma treatment process improved the interfacial bonding from the fiber pull-out test.

Yu et al. [52] developed a cementitious composite with PE fibers. The tensile strength achieved 20 MPa and the tensile strain capacity went up to 13%. Due to the hydrophobic nature of PE fibers, it was suitable for producing high-strength ECC, while the hydrophilic PVA fibers preferred the moderate strength ECC.

The tensile properties of some representative cementitious composites with PE fibers are illustrated in Table 2.

Recently, some numerical simulations were conducted on fiber-reinforced concrete (FRC) by Chalioris et al. [68]. A smeared crack model was proposed for the post-cracking behavior of fiber-reinforced concrete under tension. The FRC tension softening effect, the load versus deformation cyclic envelope and the influence of the fibers on the overall hysteretic performance were simulated by the proposed numerical model. The study revealed that FRC beams showed enhanced residual stiffness, load-bearing capacity, deformation, energy dissipation ability and cracking performance, maintaining their integrity through the imposed reversal cyclic tests [69]. Then, the smeared crack approach was adopted using special stress versus crack width relations with tension softening for the post-cracking tensile response. It captured the tension softening, the tension stiffening effect, the bending moment–curvature envelope and the favorable contribution of the steel fibers on the residual response. The results of this study revealed the favorable influence of steel fibers on the flexural behavior, the cracking performance, and the post-cracking residual stress. Beams with a higher amount of steel fibers (i.e., 1.5%) demonstrated lower deformation, increased number of cracks and decreased crack spacing and width with respect to the corresponding beams with a lower amount of fibers. The post-peak descending part of the residual stress versus strain curves depended on the volume fraction of the added steel fibers, and FRC mixtures with higher amounts of fibers exhibited higher post-cracking stress [70,71].

#### 2.2.2. Flexural Performance

Two different types of PE fibers were mixed in cementitious materials to improve the bending and impact behavior. Meanwhile, the negative effects of PE fibers on compressive strength were removed by using the hybrid system [72]. 

The bending test was conducted on PE-ECC by Said et al. [73]. The results showed that the PE fibers raised the ultimate load and deflection of the PE-ECC slab greatly. Multiple cracks were observed in the middle part of the PE-ECC slab. However, the fibers cannot be distributed uniformly in the PE-ECC, which restricted the bending behavior of it. Further, they compared the PE-ECC and PP-ECC used in a concrete slab. The result proved that PE-ECC had a better flexural performance than PP-ECC. The interface between PP fibers and the matrix was weak. Meanwhile, the tensile strength and stiffness of PP fibers were low. The PE-ECC slab showed a higher flexural strength due to its high tensile strength and elastic modulus. Hence, PE-ECC was preferred in the concrete slab.

By using PE fibers, the modulus of cementitious composites improved. The PE-ECC beam had a great modulus of rupture 27.68 MPa with a COV of 4% under bending tests. Further, the flexural deformation reached 2.5% of the span length, which showed great ductility [52]. With the bending process, a first crack appeared on the tension side at first. Afterwards, the crack number rose with the bending load while the crack width kept almost constant. Then, multiple cracks developed at the peak load. Finally, the tensile side failed since the fibers were ruptured or pulled out.

The flexural strength of PE-ECC without fly ash was 30.8 MPa, while the flexural strength decreased from 27.6 MPa to 11.5 MPa if the fly ash was incorporated. The midspan deflection was hardly affected by the amount of fly ash. Deflection went up from 4.6% to 6.3% of the span length with the increase of fly ash [54]. 

Flexural loading was imposed on PE-ECC and ECC with steel fibers. Results illustrated that the coarse and wide cracks were shown on ECC with steel fibers. Since the steel fiber had great stiffness, the matrix was easily broken. However, the PE fibers had low stiffness. The cementitious matrix would not break, which increased ductility. More small cracks were initiated in PE-ECC under bending [57].

Richardson and Coventry [74] added hybrid PP and PE fibers in cementitious composites. The dovetailed cross-section generated the gripping effect, which was good at transferring stress during loading. The flexural performance was improved by using these fibers. 

Tosun et al. [75] applied low-frequency cold plasma to treat PE fibers. The surface treatment changed the surface structures and wettability of PE fibers. PE-ECC was tested under flexural loading. The flexural results proved that plasma treatment improved the multiple cracking behavior of ECC.

PE fibers and steel fibers were added in cementitious composites, and the four-point bending test was carried out by Ahmed et al. [76]. Different ratios between PE fibers and steel fibers were evaluated. The cementitious composite with 1% steel fibers and 1.5% PE fibers had the highest flexural strength. The composite with 0.5% steel fibers and 2% PE fibers presented the highest deflection and flexural toughness. The strength loss of PE-ECC was lower than that of PVA-ECC.

The sheath/core type fibers were developed using PE as the core material, which had a two-layer structure. PE fibers were added with nanoparticles on the surface. The bond strength rose greatly, even reached hooked steel fibers. The hardness of the surface of PE fibers developed. The fiber-reinforced concrete showed ductility and strain-hardening behavior in slab tests [77].

Guerini et al. [78] compared the influence of steel fibers and polymeric fibers on the concrete, especially workability, air content, and resultant mechanical performance. Steel fibers affected the workability more adversely than polymeric fibers for a given fiber volume fraction. Both polymeric fibers and steel fibers can provide the post-cracking mechanical performances. Residual flexural tensile strength had greater variability than toughness, and similar values were obtained in both steel and macro-synthetic fibers. Under flexure, hardening behavior was observed for steel fibers, while polymeric fibers sometimes showed a softening behavior after the peak load followed by the residual strength. 

#### 2.2.3. Compressive Performance

The compressive strength of PE-ECC was lower than that of plain concrete. It decreased when the fiber usage increased [50]. The average compressive strength was 166 MPa and the corresponding compressive elastic modulus was 51.2 GPa [51]. Said et al. [73] found a similar compressive strength decrease by incorporating PE fibers. 

With the addition of PE fibers, the compressive strength decreased because of the rose of porosity. Ductile fracture mode under uniaxial compression was witnessed. Young’s modulus and strain capability increased with compressive strength, while toughness decreased. The Poisson ratio had almost no relationship with the compressive strength. The compressive strength increased with the reduction of water/binder (w/b) ratio since a lowered w/b ratio decreased porosity and led to a more compacted structure [10].

PE-ECC’s compressive strength achieved 121.5 MPa when the volume fraction of PE fibers was 2%. The average Young’s modulus was 44.26 GPa with a COV of 0.7%. It had a great modulus 44.3 GPa with a COV of 0.7%. If the aspect ratio was large and the cementitious matrix was strong, the PE fiber fractured [52]. 

Fly ash and PE fibers were added to the PE-ECC. The compressive strength dropped from 100.9 MPa to 44 MPa with a strength loss of more than 56.4% [54]. With the increase of PE fibers, the compressive strength of PE-ECC decreased since the PE fibers were difficult to be dispersed uniformly [49]. 

The reduction of the compressive strength of PE-ECC was observed by Li et al. (2020). 1% PE fibers decreased 7% compressive strength of PE-ECC compared with plain ECC. The reason was that the incorporation of PE fibers brought air voids. Hence, steel fibers were introduced in the PE-ECC to balance the negative impacts since steel fibers can prevent crack propagation, which increased the compressive strength [79]. 

Nematollahi et al. [80] studied the PE fiber reinforced geopolymer. The influence of PE fibers on strength and strain performance was investigated. Ductility was improved by using PE fibers. The constitutive law of the PE-ECC was obtained by micromechanics. PE fiber-reinforced geopolymer had greater ductility compared with PVA fiber-reinforced geopolymer from the experimental results.

Sirijaroonchai et al. [81] adopted PE fibers in high-performance cementitious composites and tested the compressive behavior. The experimental results proved that the compressive strength and the strain of the cementitious composites with PE fibers were greater than those without PE fibers. Too many PE fibers decreased the compressive strength of the cementitious composites.

Wang et al. [82] investigated the ultra-lightweight cementitious materials with PE fibers. They found that the compressive strength of the composite with PE fibers reduced dramatically.

Khan et al. [83] studied the mechanical properties of the ECC-contained concrete column under axial compression experimentally. ECC specimens showed ductile failure. A stable post-peak descending curve and higher ductility of ECC specimens were induced by the fiber bridging action. ECC confinement can change the failure mode and improve the ductility of concrete columns. Plain concrete exhibited brittle collapse when the developed tensile stress exceeded the limited strength of concrete under tension. After the peak load, the strength attenuation of the concrete specimen was fast since the concrete was brittle. In contrast, the ECC limited the failure extent and contributed to the post-peak ductility after the peak strength. The use of non-conventional mass reinforcement such as discontinuous fibers has been proved a promising alternative since the cracking of fibrous concrete requires debonding and pull-out of the randomly distributed fibers in the concrete mass. Hence, fiber-reinforced ECC demonstrated a pseudo-ductile tensile response, deformation capacity and enhanced energy dissipation capacities relative to the brittle behavior of plain concrete. Consequently, the addition of steel and PE fibers into the concrete increased mainly the strength of concrete and especially ameliorated the post-cracking behavior.

#### 2.2.4. Shear Performance

The advantageous characteristics of fiber-reinforced ECC under tension tests are also very important for the shear response of concrete structural members which is governed by the tensile response of the fibrous material. 

ECC’s strain hardening and multiple cracking behavior on the shear capacity of beams loaded in shear were investigated by experiments [84]. Results exhibited that ECC was similar to steel reinforcements for improving the shear capacity of beams. Improved shear resistance, better control of crack sizes and a more ductile shear failure were provided by ECC compared with concrete. Fibers can reduce the shear crack width and increase the crack number. The crack opening and crack sliding at failure in ECC were 20–25% of the crack size in concrete, which showed the potential of ECC exposed to moisture and other aggressive substances. ECC contributed to the shear behavior including: (1) Fiber bridging of shear cracks, increasing the shear ability; (2) Traditional shear reinforcement was activated at smaller crack deformations; and (3) Crack opening was restricted by fiber bridging mechanism and by activating traditional shear reinforcement at smaller crack deformations. The shear crack development mechanism of reinforced ECC included 5 stages: (1) Crack initiation without noticeable sliding, which was restricted due to fiber transferring stresses across the crack; (2) Crack sliding and opening, which was restrained by fiber bridging, aggregate interlock and stirrups. When the crack opening was greater than one-half of the maximum aggregate size, the aggregate interlock stopped. The limited crack deformation was caused by the enhanced crack control by fibers, which transferred stresses over the crack with a small crack opening. However, traditional reinforcement needed greater deformation to activate the stress transferring over the crack; (3) Crack opening and sliding developed slowly due to the combined bridging effect by fibers and stirrups. The aggregate interlock was reduced since the crack opening was great in this stage. The stirrups and longitudinal reinforcement fully worked. Additional transverse reinforcement prevented cracking due to the increased cross-sectional area bridging the crack; (4) Increased rate of crack deformation. Crack opening and sliding were limited mainly by stirrups. The stirrups carried increasing stress and ultimately yielded. Fibers reached maximum bridging stress and failed. At the end of this stage, force led to pullout and rupture of the fibers at the weakest crack; (5) Rupture of stirrups and fibers in ECC resulted in the final failure of specimens.

Overall, steel fibers generated heightened shear properties in the concrete mixes, which were similar to PE fibers [85]. Recent studies by Chalioris et al. [86,87,88] indicated that fibrous beams displayed strengthened shear properties with higher shear strengths, enhanced absorbed energy ability and enhanced crack patterns since fibers can bridge the shear crack, enhance the shear ability and prevent the brittle failure. Results also showed that fiber can be adopted to replace minimum stirrups. 1.2% fiber percentage can change the failure mode of specimens from shear failure to flexural failure [89].

### 2.3. Performance under Dynamic Loading

#### 2.3.1. Impact Loading

Maalej et al. [90] investigated ECC with PE fibers and steel fibers under dynamic tensile loading and impact loading. ECC showed higher tensile strength and strain capacity under dynamic loading compared with plain concrete. The strain-hardening effect worked well under dynamic loading. Tight cracks about 0.1 mm were observed in the test, which was similar to the result under static loading. By distributing cracks uniformly, PE-ECC showed great potential as the protective material.

**Figure 2:**
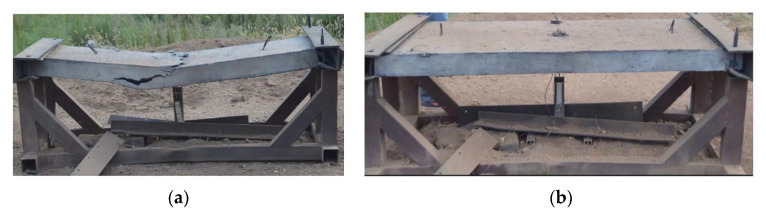
The detonation of (**a**) normal concrete; (**b**) PE-ECC [91].

Li et al. [91] applied PE fibers, steel fibers and steel wire mesh to reinforce the concrete slab under blast loading. The in situ blast experiment was carried out as depicted in Figure 2. After close-in blast detonation, the reinforced concrete slab was almost intact. No macrocrack or deflection was witnessed, while the shear crack and flexural response appeared in the sample without PE fibers. The ductility and tensile resistance were enhanced due to the combination of PE fibers, steel fibers and the steel wire mesh.

Previously, the cementitious composites with 0.5% PE fibers and with hybrid fibers (steel fibers and PE fibers) were investigated under impact loading. The experimental result showed that the raised strain rate increased the compressive strength, ultimate deformation, elastic modulus and fracture toughness of cementitious composites [92]. Similarly, Soroushian et al. [93] adopted PP and PE fibers to produce the cementitious composite. Experimental results proved that hybrid fibers increased impact loading resistance and toughness.

The loading rate effect influenced the properties of PE-ECC greatly from previous research. The hybrid fibers (1.5% PE fibers and 0.5% steel fibers) were adopted in the ECC. It can be concluded that the tensile strength rose with the strain rate. The strain-hardening behavior and energy absorption ability were enhanced with PE fibers. PE fibers can dramatically improve strain capacity, which was required for impact-resistance structures. Spalling and fragmentation were reduced greatly [90,94]. 

The dynamic compressive performance of cementitious materials with PE fibers and steel fibers was tested. The higher strain rate led to a greater compressive strength of the cementitious composites. The steel fibers failed from pull-out failure to fracture with the increasing loading rates. More steel fibers were fractured at higher loading rates [95].

The strain rate effect of cementitious materials with PE fibers was tested by Wang et al. [92]. The experimental result showed that the loading rate had little influence on the compressive strength and elastic modulus of it. The compressive strength and elastic modulus did not change greatly when the strain rate ranged from 40/s to 300/s. A higher dynamic toughness was witnessed when hybrid fibers were used in cementitious composites. 

PE fibers were mixed with the densely packed ultra-high-strength matrix to produce the cementitious composite. Different tensile strain rates from 10^−4^/s to 10/s on the cementitious composite were investigated. The first crack initiation stress and tensile strength of the cementitious composite increased by about 53% and 42%, respectively. No chemical bond between the PE fiber and the matrix existed, which made the composite insensitive to loading rates [96]. Maalej et al. [90] also observed the negligible strain-rate effects of the tensile strain capacity of cementitious composites with PE fibers and steel fibers. However, tensile strength rose greatly.

Yang and Li [97] studied the dynamic properties of cementitious composites with PE fibers and the ultra-high strength matrix. They used the PE fibers to reduce the interfacial chemical bonding between the cementitious matrix and PE fibers, while the fiber bridging effect was ensured. In this way, the cementitious composite was less sensitive to the loading rate. 

Curosu et al. [65] used the Split Hopkinson bar to test the cementitious composite with PE fibers. Young’s modulus, tensile strength and fracture energy all went up under dynamic loading. Strain capacity decreased. Meanwhile, more fibers were fractured under dynamic loading than under static loading. Multiple cracking behavior of composites was noticed under dynamic loading.

Considering that PE fibers had a great property in the pull-out test compared with steel fibers, PE fibers offered an improvement in the impact resistance of ECC. Previous research proved that PE fibers provided the ECC a better ductility compared with steel fibers under impact loads. The reason was that the PE-ECC had more dispersed microcracks [98].

Ohtsu et al. [99] added the PE fibers into cementitious materials and found that the spall failure of the civil engineering structure reduced under blasting. The fractured area decreased due to the PE fibers, which increased flexural toughness.

#### 2.3.2. Cyclic Loading

With polymeric fibers, ECC can improve the performance of columns under cyclic loading. Zhang et al. [100] studied the seismic behavior of reinforced engineered cementitious composite (RECC) short columns and H-steel reinforced engineered cementitious composite (SRECC) short columns by combined axial compression and transverse cyclic loads. The failure behavior of the RECC column and SRECC column was controlled by shear, and the SRECC column had a bond failure. However, the plastic deformation capacity of the SRECC column was much higher than that of the RECC column. The development mechanism of shear cracks in RECC included the following stages: before the formation of the main diagonal shear cracks, the stirrup stress was very small, and the shear force was mainly borne by the arch (concrete/ECC column). After the main diagonal shear cracks were formed, the stirrup stress increased rapidly, and the shear contribution of the truss action increased. For concrete specimens, due to the softening of the concrete diagonal strut, the shear contribution of arch action decreased. The aggregate interlocking force on the crack surface decreased rapidly after the stirrups yielded. For ECC specimens, the fiber bridging force can effectively delay the propagation and widening of diagonal cracks, thus delaying the softening of the ECC diagonal strut. The fiber bridging force between diagonal cracks can transfer the tensile stress in the circumferential direction until ECC reached the ultimate tensile strain. The results showed that the shear strength of ECC specimens was 23% higher than that of concrete specimens. 

Li et al. [101] investigated the mechanical behavior of ECC-strengthened columns under lateral cyclic loading. The results showed that all repaired columns were broken in the ductile flexural manner. Spalling of ECC and exposure of longitudinal reinforcements were not found in the test. There were many fine cracks on the surface of the ECC jacket. With a thicker ECC jacket, the local lateral expansion of the ECC jacket can be effectively mitigated. ECC produced a strong lateral restraint. According to the hysteretic responses and the cracking behavior of specimens with fibers, the cracking can be delayed and the flexural/shear ductility can be improved compared with the behavior of specimens without fibers. Thus, due to the tensile stress transfer capability of the fibers across crack surfaces (known as crack-bridging) and also to the fact that fibers provided significant resistance to shear across developing cracks, the ECC specimen had excellent hysteresis response. The hysteresis loops were plump. The strength reduced slowly. Meanwhile, the absorbed energy capacities increased. With the transferred stress by fibers across crack surfaces, the failure mode changed from the brittle failure to a ductile one. This is good for building structures resisting a strong earthquake. 

These phenomena also existed in steel fiber-reinforced concrete. Chalioris [102] found that steel fibers prevented crack propagation and the closure of cracks during reversal loading during cyclic tests. Fiber-reinforced concrete can support cyclic loading conditions and display greater energy dissipation capacity compared with plain concrete. Later, they also found that 3% was the optimal fiber dosage for concrete which showed an improved cyclic response [103]. No inclined cracks of shear nature or concrete brittle spalling appeared along the length of the beam. The structures failed due to ductile flexure. 

### 2.4. Performance under Different Temperatures

**Figure 3:**
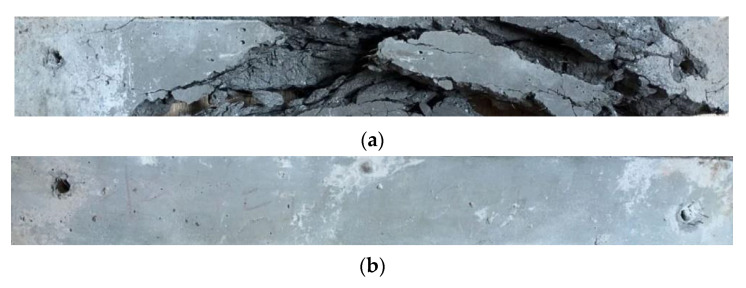
The influence of high temperatures on (**a**) concrete; (**b**) PE-ECC [79].

The PE-ECC specimen was tested after heat treatment. It showed little spalling while the ECC with only steel fibers had an obvious spalling, as was exhibited in Figure 3. It illustrated that PE fiber can release pore pressure to avoid the spalling of the cementitious matrix. Steel fibers cannot stop the rise of pore pressure [79]. Meanwhile, PE fibers had a temperature softening effect [104].

The flexural properties of PE-ECC were significantly affected by the temperature. At temperatures lower than 400 °C, the post-peak load and toughness increased. However, the load-deflection curve dropped greatly due to the evaporation of PE fibers when the temperature was greater than 400 °C [105].

Since the melting point of PE fibers was low, the working temperature was important and investigated by Liu and Yu [106]. The critical temperature was influenced by the glass-transition temperature. The suggestion of the working temperature was 70 °C, below which the mechanical properties were guaranteed.

Yun [107] studied the mechanical properties of ECC with hybrid PVA and PE fibers under freezing and thawing cycles. It can be concluded that the hybrid system had more benefits in mechanical properties than ECC with PE fibers alone. Three hundred freezing–thawing cycles had little effect on the compressive properties and modulus of ECC. The flexural deformation was reduced by using the hybrid system.

### 2.5. Microstructure of PE-ECC

It can be observed in the SEM that the PE fibers in PE-ECC were damaged greatly in the pull-out process. Some PE fibers broke directly due to the strong interfacial interaction between PE fibers and the cementitious matrix. A great number of PE fibers were pulled out from the cementitious matrix. Fibrillation was witnessed. Some fibers were broken during extrusion, which proved that PE fibers worked well in PE-ECC and bore great loads [52,54]. 

**Figure 4:**
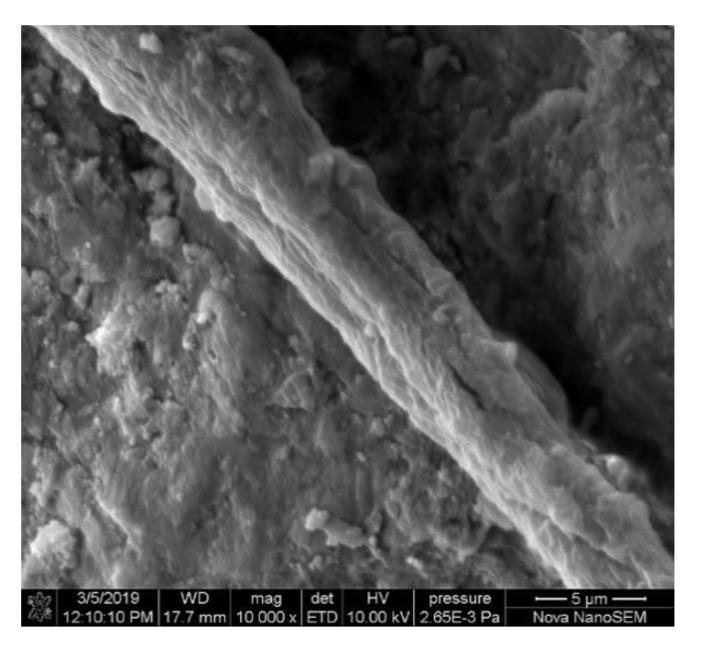
SEM micrograph of a PE fiber in cementitious composites [108].

PE fibers had a smooth surface in cementitious composites. However, the rough surface of PE fibers was witnessed in our previous research as presented in Figure 4 [108]. The PE fibers were damaged during the pull-out process since the fiber/matrix bond was strong. Mechanical interaction rather than chemical bonding between PE fibers and the matrix existed. The lateral figure of PE fibers also proved its strong bonding due to the scratch on the surface of PE fibers. The necking behavior of PE fibers happened during the pullout process in our previous research. The cross-section became small from SEM experiments, which illustrated strong bonding between the cementitious materials and PE fibers. The strong bonding increased the bridging effect, which meant multiple cracks were initiated and energy could be dissipated significantly. Good bonding also meant the strain hardening effect of PE-ECC, since the material could bear more of a load even after the cracks were initiated. In order to strengthen the interfacial bonding, a denser ITZ with a lower void ratio was desired.

## 3. The Properties of Structures Repaired by PE-ECC

### 3.1. Column

Gholampour et al. [109] added PE fibers into concrete to repair an aged concrete column. The concrete column with a PE-ECC jacket had ductility under compression, while others without PE fibers showed brittle behavior. The larger volume fraction of PE fibers increased the ductility of the repaired system. The repairing mechanisms contained two categories. On one hand, the PE-ECC increased the cross-section of the concrete column. On the other hand, the PE-ECC provided lateral confinement, which can enhance the load-carrying ability of existing columns.

Some other research used PE to wrap the damaged column for the repairing goal [110]. The ultimate load of the repaired column was 48.04% greater than that of control samples. The deformation of the repaired column was restrained due to the PE sheet. Cracks were also restricted. The PE sheet proved to be effective in carrying additional loads.

### 3.2. Beam

Two types of repairing methods of damaged beams (i.e., patching and layering) were investigated by Kim et al. [111]. PE fibers and PVA fibers were adopted in the cementitious materials as the components of repairing materials. Results showed that ECC-repaired beams had greater first crack initiation load and peak load under shear tests. The weak point of the repaired beam was the interface between the beam and the repairing composite. The tensile properties of PE-ECC influenced the peak load, the stiffness and the ductility of the beam when the patching method was used. However, the delamination properties of ECC were more important when the layering method was applied.

The RC beam was repaired by PE-containing cementitious composites. Using the bending test, the failure mode of the patching material was recorded and was different from the tensile tests [46]. Cracks of PE-ECC existed only close to existing cracks of RC beams in bending tests. The crack opening and crack patterns obtained from the zero-span tensile tests were similar to those of the repaired beam specimens.

Hussein et al. [112] developed a steel/PE-ECC repairing system for damaged concrete beams. The combination of steel bars and PE-ECC can remove the strain localization and delay the cracking of PE-ECC. The average strain of PE-ECC with steel bars was 3.76 times that of an unreinforced PE-ECC layer. The ductility increased significantly. The initiated cracks in the PE-ECC were more uniform, which avoided the brittle failure of the concrete beam.

The combined PE and PVA fibers were mixed in the ECC to repair the concrete beams exposed to freeze–thaw cycles by Yun [107]. The freeze–thaw process had little effect on the compressive strength of ECC, while it influenced the flexural and tensile performance. The repaired concrete beam had significantly higher strength and ductility. With the increase of laying thickness, the strength and ductility improved since the kinking occurred. The crack distribution of the beam was also influenced by the repairing material. Hence, the brittle failure was delayed.

Hybrid PE and PVA fibers were used in cementitious materials to repair the concrete beam. The flexural performance of the repaired beam was investigated. ECC can improve the ultimate load of beams under bending. The yielding time was put off. PE fibers contributed significantly to the ductility improvement. Concrete crushing and spalling phenomena were prevented by the repairing material. Meanwhile, the repaired concrete beams by ECC had better energy dissipation ability and lower stiffness reduction after cyclic loading. The cracks of the concrete beam repaired by ECC were much finer than that without repairing [113].

Previously, the interface between the damaged structure and the repairing material should be tough to enhance the interfacial bonding and the repairing effect. Kamada and Li used PE-ECC to repair the concrete and tested the flexural behavior [114]. When the surface was tough, the repairing effect was worse than the smooth surface situation. The phenomenon was different from repairing using the concrete healing agent. Meanwhile, 0.28 was the best water-binder ratio (w/b) when PE-ECC was adopted as the repairing material. 

Concrete beams with fire damage were repaired by PE-ECC. Then, those beams were tested by 4-point bending tests. The results illustrated that the repaired beams had more uniformly distributed cracks, greater strength and higher displacement at peak load compared with the control samples, which showed the ductile failure mode [115].

Lim and Li [36] applied PE-ECC as the repairing material to heal aged concrete beam. The trapping mechanism of PE-ECC was applied to analyze the effective repairing behavior. The repaired structure showed higher strength, greater ductility and energy-absorbing behavior. The cracks of PE-ECC were multiple with a small crack width, as desired by civil engineers. The reason for the trapping mechanism was the rise of the toughness of the PE-ECC. At first, the PE-ECC had a low toughness, which can attract the interfacial crack, and kinking cracks were observed. After the interfacial cracks propagated into the PE-ECC, the PE fibers played an important role in arresting cracks since it had a high toughness. After the kinked crack was in traps, the interfacial crack propagated along with the interface and fell in the next trap by PE fibers. In this way, the delamination phenomenon was avoided by using the PE-ECC material. The PVA-ECC and ECC with steel fibers were designed, and the same experimental configuration was imposed on them. The comparison test proved that PE-ECC had the highest tensile strength [116]. 

Yun et al. [117] adopted PE-ECC to repair a coupled beam shear wall under earthquakes. Steel cords were added to the repairing material. Due to the bridging effect of PE fibers, the cracks were finer and distributed in a wider area. The ductility and strain-hardening effect were obvious during the test under high shear loading. The seismic resistance of the beam rose. 

Kobayashi and Rokugo [118] developed a new technology for repairing concrete beam. A PE fiber net was applied on the surface of the damaged concrete structure fora repairing purpose. Meanwhile, PE fibers were mixed in the cementitious material. The bending test results proved that multiple fine cracks appeared rather than a single big crack because of the combined use of the PE fiber net and ECC. When the yielding point came, the crack width increased. The patching prevented rebar corrosion due to these small cracks. 

Zhang and Li [119] investigated the deformation of beams repaired by PE-ECC, PC and SFRC. The kinking crack preferred a smooth interface between PE-ECC and base concrete. PE-ECC/PC system presented a higher peak load and corresponding deflection compared with other repairing materials. 

Maalej et al. [120] adopted PE/steel fiber reinforced cementitious material to repair a corroded beam. The repaired cementitious composite was installed at the tensile zone of the beam. The normalized frequency of the beam was considered to evaluate the health condition of the beam. After repair, the normalized frequency decreased significantly due to the low stiffness of ECC and the weak bonding between the aged beam and the repairing material.

The corrosion protection ability of PE-enabled concrete as a repair material was investigated. After the PE-enabled concrete repaired the normal concrete, chloride penetration depth was reduced and reinforcement corrosion was avoided, which illustrated its good chloride-resistant ability. The best fiber content was 0.75% as a repair material [121].

Corroded steel bars may cause danger in structures. Chen et al. [64] presented PE-enabled cementitious materials as a repairing material to cure the steel corrosion of concrete. Then, a four-point bending test was carried out. Results showed that the tensile strength reached 8 MPa and the ultimate tensile strain was 2%. Further, the PE-ECC material can fully recover the load-carrying capacity of the corroded specimen.

### 3.3. Slab

Yun et al. [122] applied PE-ECC to strengthen a concrete frame by building an infill wall in the concrete structure. ECC contained 0.75% PE fibers as the volume fraction. The infill wall by PE-ECC showed the ductile failure mode, while the infill wall by concrete displayed brittle failure. The shear strength of the PE-ECC wall was two times the shear strength of a normal concrete infill wall. Meanwhile, the deformation ability did not drop.

Maalej et al. [123] used hybrid fiber ECC containing 0.5% steel and 1.5% PE fibers to repair masonry walls. After the repair, a patch load, uniformly distributed load and low-velocity projectile impact were imposed on the masonry walls. The experimental results illustrated that the repaired wall had good out-of-plane resistance. The ultimate strength increased dramatically. The deflection capability rose to a maximal 22 times compared with the control sample. Meanwhile, the strength of masonry walls under impact was raised, and fewer fragmentations were witnessed.

Kesner and Billington [124] developed a retrofitting system for aged critical structures. The PE-ECC was adopted as the main material for panels instead of traditional concrete. The PE-ECC showed great tensile and compression capacity. By changing the number of PE-ECC panels, the desired strength, stiffness and energy dissipation can be obtained. 

Yun et al. [125] used PE-ECC to repair the one-way slab with a different thicknesses of ECC. Expansive admixtures were added into ECC. The flexural and compressive strength increased up to 1.07 and 1.08 times compared with the ECC without expansive admixtures. The tensile strength decreased slightly. The flexural strength of the one-way slab rose due to the repairing layer ECC. The stiffness increased at the same time. The expansive admixture contributed to the higher stiffness and crack initiation load. Multiple fine cracks were observed in the bending test instead of a wide single crack. The thickness of the repairing layer on the tensile surface influenced the repairing effect.

## 4. The Self-Healing Effect of Cementitious Composites with PE Fibers

In concrete structures, cracks cannot be avoided from damage, shrinkage, creep and corrosion. Fortunately, PE-ECC has the property of self-healing, which can remove these cracks automatically. Using a self-healing effect can lower maintenance costs, increase service life and reduce manual repair. Self-healing concrete contains two categories: autogenous healing and autonomous healing. PE-ECC belongs to autogenous healing due to the carbonation of calcium hydroxide and continuing hydration of cement grains, while PE fibers can restrict the crack width [126]. 

Li and coworkers firstly developed PE-ECC that can heal cracks itself. The PE fibers can decrease the crack width, and further hydration can cause the self-healing effect of PE-ECC [127]. The average crack width was less than 50 μm in the PE-ECC, which facilitated the self-healing effect.

Later, many other scholars confirmed the self-healing behavior of PE-ECC and investigated the healing efficiency of PE-ECC [128]. PE-ECC was found to have high ductility and multiple cracks, which was different from plain concrete. The self-healing products on the surface of cracks were calcium carbonate, which recovered tensile strength and decreased water permeability. 

Mihashi et al. [129] investigated the durability of PE-ECC and ECC with PE and steel fibers. The experimental results proved that hybrid fiber reinforced concrete was better than plain concrete and PE-ECC due to a small crack width which reduced steel corrosion and led to the self-healing effect.

Koda et al. [130] tested the self-healing effect of ECC with different kinds of fibers. The results showed that when the crack width was less than 100 micrometers, the self-healing efficiency caused by PE fibers and PVA fibers were similar.

Nishiwaki et al. [131] investigated the self-healing effect of PE-ECC. Water-tightness and mechanical properties were tested. The hybrid fibers (i.e., steel fibers and PE fibers) proved to be effective in self-healing considering that mechanical properties and water-tightness increased. Fine cracks were observed as healed using hybrid fibers.

Kunieda et al. [132] used a high strength matrix and 88 GPa high-stiffness PE fibers to produce PE-ECC, which exhibited a good self-healing effect. The crack width in the research was less than that of normal ECC due to the strong restriction by PE fibers.

Previous research compared the self-healing ratio by alkali-activated slag-based composites and cementitious composites using PE fibers. The results illustrated that cementitious materials had a higher healing recovery than alkali-activated slag-based materials. Meanwhile, calcium carbonate was the filling material in cracks of PE-ECC [133].

## 5. Conclusions

This literature review systematically has revealed the performance of PE fibers-reinforced cementitious composites in civil engineering to apply these characteristics to structures. Repaired concrete structures by cementitious composites with PE fibers have also been investigated. The following conclusions are drawn.

As a repairing material, PE-ECC has great potential due to its outstanding properties in structural retrofitting, especially when exposed to moisture, impact loading, aggressive substances, large inelastic deformation and other harsh environments. The tensile strength, flexural strength, fatigue performance, ductility, strain-hardening performance, post-peak mechanical properties and impact resistance of PE-ECC are better than those of plain concrete. Cracks in PE-ECC are distributed uniformly with a small width. The energy absorption of PE-ECC is improved compared with plain concrete. It is effective in resisting blast load and temperature influence. Interfacial bonding between PE fibers and the cementitious matrix has a good property. The interfacial bonding between PE fibers and the cementitious matrix is mainly mechanical since it is hydrophobic, which can increase energy absorption. The addition of PE fibers into the concrete increases mainly the tensile strength of concrete and especially ameliorates post-cracking behavior. Fibers have been proven to be a promising non-conventional reinforcement in shear-critical concrete beams. Based on the review, larger aspect ratio, higher fiber content and greater cement contents increase flexural/shear strength, deflection at ultimate load and elastic modulus. However, when the fiber content or aspect ratio rises, the fresh matrix is difficult in dispersing the PE fibers, resulting in low workability and the non-homogeneous matrix. Meanwhile, an increase in PE fiber content induces an undesirable orientation of fibers.

The mechanical properties of PE-ECC repaired structures are reported. Based on the presented literature review, the experimental results prove its potential as a repairing material for beams, columns and slabs. The repaired structures exhibit a greater ductility, impact resistance, fatigue performance, strength, strain-hardening effect, multi-crack behavior and energy dissipation capacity. Delamination and spalling can be avoided by using PE-ECC as the repairing material due to the kink-crack trapping mechanism or the formation of a large plastic zone in the PE-ECC layer.

However, the existing literatures report that the incorporation of PE fibers increases the air void, which has a negative effect on the ITZ of ECC. Meanwhile, homogeneity should be optimized, which challenges the polyethylene fiber reinforced cementitious composites. In this regard, the manufacturing methods of PE-ECC should be improved for practical use. A new manufacturing method to produce PE-ECC with a low void ratio is needed and will be investigated in our future work. Additional increases in failure ductility of ECC would be possible by using fibers that can resist higher deformations and engineering the composite to fail by fiber pullout rather than rupture.

## Figures and Tables

**Table 1:** The physical properties of polyethylene (PE) fibers [39,40,41].

Performance	PE Fibers
Tg (°C)	−133–100
Tm (°C)	105–140
Density (g/cm^3^)	0.92–0.96
Water absorption (%)	0–0.2
Heat deflection temperature (°C)	32–60
Coefficient of thermal expansion (mm/mm/°C×10^5^)	10–13
Tensile strength (MPa)	14.5–600
Elastic modulus (GPa)	0.055–31
Elongation (%)	2–800
Impact strength (J/m)	>26.7
Diameter (μm)	10–1000
Relative adhesion to matrix	good
Relative alkaline stability	Excellent

**Table 2:** The tensile properties of cementitious composites with PE fibers.

Literature	l/d	Volume Fraction of PE Fibers (%)	Tensile Strength (MPa)	Tensile Strain (%)
[46]	500.0	1.5	10	2.8
[49]	500.0 or 315.8	1–2.5	4.2–5.3	2.5–5
[52]	900.0	2	17.8	8.5
[64]	500.0	2.2	10.8	2.4
[51]	453.6	2	14.5	3.4
[65]	600.0	2.1	6.5	5.7
[66]	1500.0	1.75	8.8	7.2
[67]	1500.0	1.75	13.1	7.5
